# Inter-Facility Emergency Department Transfers for Non-Contracted Insurance Status: Disproportionate Impact Upon Minority Patients

**DOI:** 10.5811/westjem.48502

**Published:** 2025-07-18

**Authors:** Andrew Holzman, Malik Aaron, Krish Nayar, William Rankin, Melissa Tapia, Douglas Rappaport

**Affiliations:** * Mayo Clinic Hospital Alix School of Medicine, Phoenix, Arizona; † Mayo Clinic Hospital, Phoenix, Arizona; ‡ Mayo Clinic Hospital, Department of Emergency Medicine, Phoenix, Arizona

## Introduction

We examined inter-facility transfers due to non-contracted insurance status from the Emergency Department at a large tertiary care center in the Phoenix metropolitan area. This would go in results and conclusion, not introduction.

Transfers between Emergency Departments can have an important impact on patient care, with inter-hospital transfer associated with higher cost, longer overall length of stay, lower odds of discharge home, and higher risk of 3 and 30-day mortality for certain conditions.

Although such transfers are regulated by the Emergency Medical Treatment and Active Labor Act (EMTALA), transfers remain possible after patients have been stabilized, and evidence suggests that uninsured patients are more likely to experience inter-hospital transfer from the Emergency Department. We hypothesize that patients from minority populations will be disproportionately impacted by transfers for non-contracted insurance status from the Emergency Department.

## Methods

Data were extracted from the hospital’s electronic health record system, EPIC. The study period covered Emergency Department visits from January 1, 2021, to December 31, 2023, and records for all patients who presented to the ED during the study period were queried. Records were excluded if final disposition was not admission to an in-facility floor, the in-facility observations unit, or a facility transfer.

Records for patients who underwent facility transfer were reviewed to determine which transfers were due to insurance contracting status. Patients transferred to access care not provided at our institution were excluded.

The number of patients transferred for insurance incompatibility was compared with the number admitted either to observation or inpatient status at our facility for groups with socioeconomic minority status.

## Results

We identified 336 transfers due to insurance incompatibility. Among patients transferred for non-contracted insurance status, the most common insurance type was Medicare Advantage plans, with 194 (53%) patients transferred.

We found significantly increased transfer rates due to insurance incompatibility for Hispanic patients (1.31% of all patients either admitted or transferred compared to 0.87% for non-Hispanic whites; Odds Ratio (OR) 1.52 give 95% confidence intervals for all odds ratios, p= .0036) and non-English speakers (2.06% compared to 0.90% for English speakers; OR 2.32, p < .001). For the combined group of non-White, Hispanic, or non-English speaking, patients the rate of transfers due to insurance was 1.30% (compared to 0.84% for White, non-Hispanic English speakers; OR 1.55 p=.0001).

## Conclusions

Our data suggest that hospital insurance contracting policies disproportionally affect minority groups, who may be more likely to hold non-contracted insurance classes. Further research is needed to determine the impact of limited provider networks associated with some health insurance plans. Hospital networks and health insurers alike must find solutions to address these systemic barriers to equitable care for minority and non-English speaking populations.
